# Patient-controlled intravenous analgesia with tramadol and lornoxicam after thoracotomy: A comparison with patient-controlled epidural analgesia

**DOI:** 10.1097/MD.0000000000014538

**Published:** 2019-02-15

**Authors:** Juying Jin, Su Min, Qibin Chen, Dong Zhang

**Affiliations:** Department of Anesthesiology, The First Affiliated Hospital of Chongqing Medical University, Chongqing, China..

**Keywords:** analgesia, lornoxicam, pain, patient-controlled, postoperative, thoracotomy, tramadol

## Abstract

To determine efficacy and safety of patient-controlled intravenous analgesia (PCIA) with tramadol and lornoxicam for postoperative analgesia, and its effects on surgical outcomes in patients following thoracotomy.

The records of patients who underwent thoracotomy for lung resection between January 2014 and December 2014 at our institution were reviewed. The patients were divided into 2 groups according to postoperative pain treatment modalities. Patients of the patient-controlled epidural analgesia (PCEA) group (n = 63), received PCEA with 0.2% ropivacaine plus 0.5 μg/mL sufentanil, while patients in the PCIA group (n = 48), received PCIA with 5 mg/mL tramadol and 0.4 mg/mL lornoxicam. Data were collected for the quality of pain control, incidences of analgesia related side effects and pulmonary complications, lengths of thoracic intensive care unit stay and postoperative hospital stay, and in-hospital mortality.

Pain at rest was always controlled well in both groups during the 4-day postoperative period. Patients in the PCIA group reported significantly higher pain scores on coughing and during mobilization in the first 2 postoperative days. The incidences of side effects and pulmonary complications, in-hospital mortality and other outcomes were similar between groups.

PCIA with tramadol and lornoxicam can be considered as a safe and effective alternative with respect to pain control and postoperative outcomes for patients underwent thoracotomy.

## 1 Introduction

Thoracotomy is well recognized as one of the most painful surgical procedures. Adequate pain relief after thoracic surgery is of particular importance, not only for keeping the patient comfortable but also for reducing the incidence of postoperative complications.^[1]^ Compared to systemic opioids, thoracic epidural analgesia (TEA) with local anesthetic and opioid has been demonstrated to provide superior post-thoracotomy pain management with minimum impairment of pulmonary function.^[2,3]^ However, reduced pulmonary morbidity is primarily seen only in high-risk thoracic patients. So far, the benefits of TEA on the length of hospital stay and the in-hospital mortality are still controversial.^[4,5]^ On the other hand, epidural catheterization may require additional anesthesia preparation time and may fail, may be contraindicated as well as associated with rare, but serious complications.^[6,7]^ Therefore, intravenous analgesia is also widely used for pain control after thoracotomy due to its technical simplicity and safety.

Tramadol is a centrally acting analgesic with 2 different mechanisms of action. One enantiomer exerts a predominantly weak μ opioid effect, whereas the other inhibits norepinephrine and serotonin reuptake, activating descending monoaminergic inhibitory pathways.^[8]^ Tramadol has been shown to offer a similar analgesic potential to opioid in patients receiving patient-controlled intravenous analgesia (PCIA) following thoracotomy. Its relative lack of sedative and respiratory depressive effects makes it especially suitable for analgesic use after thoracic surgery.^[9]^

Lornoxicam, a member of the oxicam group of nonsteroidal anti-inflammatory drugs (NSAIDs), was reported to improve postoperative pain control and decrease tramadol consumption as well as the incidence of side effects when administered with tramadol by PCIA after abdominal surgery.^[10,11]^ Efficacy and safety of intravenous application of tramadol with NSAIDs have been documented in patients who have undergone the thoracic procedure.^[12,13]^ However, no study has looked at post-thoracotomy pain control as well as postoperative outcomes of PCIA with tramadol and an NSAID compared to patient-controlled epidural analgesia (PCEA).

At our hospital, as an alternative technique to PCEA, tramadol has been administered in combination with lornoxicam by PCIA for pain management following thoracic surgery. Therefore, we performed this retrospective study primarily to examine the differences in postoperative pain intensity and side effects between patients who received PCIA or PCEA after thoracotomy for lung resection. A secondary aim was to determine whether postoperative outcomes differed significantly between the 2 analgesia regimens.

## 2 Materials and methods

Following the approval from our hospital's Ethics Committee (registration number: 2013-61), we reviewed the medical records of 124 consecutive patients who underwent elective posterolateral thoracotomy for lung resection between January 2014 and December 2014 at our institution. After excluding patients under 18 years of age, patients with a chronic pain condition or with regular consumption of opioids for more than 2 weeks preoperatively, and those had their epidural catheter dislodged within the first 2 days after surgery, the remaining 111 patients were included in this study. The patients were divided into 2 groups according to their postoperative pain treatment modalities. Patients in the PCEA group (n = 63), received PCEA with ropivacaine plus sufentanil, while patients in the PCIA group (n = 48), received PCIA with tramadol–lornoxicam combination.

The decision for postoperative analgesic modality was based on the outcomes of preoperative evaluation, and conversation between the attending anesthesiologist and patient about benefits and risks of both techniques. All patients gave written consent for the analgesic technique that was to be used.

In the PCEA group, a thoracic epidural catheter was placed at the level of T5-T7 using a loss of resistance technique before the induction of general anesthesia. The catheter was inserted cephalically 4 to 5 cm and fixed in place. A 3 mL test dose of 2% lidocaine was administered to exclude accidental intrathecal injection. Then an additional 1% ropivacaine 5 mL was administered. The level of sensory block was aimed at T2-T10, tested by pinprick method 20 minutes later. Epidural administration of 1% ropivacaine 5 mL was repeated every 60 minutes during surgery.

General anesthesia was induced with 2 to 3 mg/kg of propofol, 2 to 4 μg/kg of fentanyl, and a nondepolarizing muscle relaxant, either vecuronium (0.1 mg/kg) or atracurium (0.5 mg/kg) was used to facilitate endotracheal intubation with a double-lumen tube, and the lungs were ventilated with a mixture of 50% oxygen/air. Anesthesia was maintained with sevoflurane, fentanyl, and remifentanil as necessary. After completion of surgery, the double-lumen tube was removed and the patient was shifted to the postanesthetic care unit (PACU) for close observation for at least 2 hours, and then transferred to the thoracic intensive care unit (TICU).

Postoperative pain treatments were initiated at the end of surgery. In the PCEA group, patients received a 0.2% ropivacaine solution with 0.5 μg/mL sufentanil. The epidural infusion was delivered at a rate of 3 to 5 mL/h with a 2 to 3 mL bolus and 20-minutes lockout interval. Patients in PCIA group received a 10-mL combination of 5 mg/mL tramadol and 0.4 mg/mL lornoxicam as a loading dose, followed by a continuous infusion at a rate of 1 to 2 mL/h, and a bolus of 1 to 2 mL with a lockout interval of 15 minutes.

The acute pain service (APS) is responsible for postoperative pain management at our Hospital. The visual analog scale (VAS) was used for assessing the degree of pain relief during patient controlled analgesia (PCA) treatment. The VAS ranged from 0 = no pain to 10 = unbearable pain. The nursing staff in the surgical ward measured pain intensity every 4 hours while the PCEA or PCIA was in use. The APS routinely reviewed each patient 2 times per day, and whenever necessary. VAS scores at rest, on coughing, and during mobilization (defining as changing body position) were recorded. If patients reported a pain score at rest equal to or greater than 4 on 2 consecutive time points, the ward nurse paged an APS member, who then visited the patient and adjusted the PCA settings as needed until the patient had a pain score at rest of less than 4, which was considered adequate analgesia. Supplemental morphine 5 to 10 mg was administered intravenously if the adjustment of the infusion pump still resulted in insufficient pain relief. Side effects of analgesia were also assessed daily. Antiemetic or antipruritic drugs were prescribed on an as-required basis. Ondansetron 4 to 8 mg was intravenously administered every 12 hours to treat nausea and vomiting. Intravenous diphenhydramine 20 mg was used every 12  hours for pruritus. The PCEA or PCIA protocol was maintained until patients were switched to oral analgesics. All patients were questioned as to their satisfaction with the quality of analgesia using a 4-point scale (0 = very unsatisfied, 1 = unsatisfied, 2 = satisfied, and 3 = very satisfied) at the end of PCA treatment period.

Patients’ charts were analyzed for demographic data including gender, age, height, weight, American Society of Anesthesiologists (ASA) status, preoperative forced vital capacity (FVC) and forced expiratory volume in 1 second (FEV1), type and duration of surgery. Information about the maximum VAS values at rest, during coughing and mobilization in PACU, for the day of surgery (POD 0, from arrival in the TICU) and for postoperative days 1 (POD 1) through 4 (POD 4), and side effects of each analgesic modality including nausea and/or vomiting, pruritus (localized or generalized itching with or without treatment), oversedation (difficulty arousing the patient verbally), urine retention, hypotension (systolic blood pressure <90 mm Hg or >20% decrease from baseline), and respiratory depression (less than 9 breaths/min or apnea requiring naloxone and medication dose adjustment) were also collected. Additional parameters included requirement for supplemental intravenous morphine, duration of PCA use, patient satisfaction, occurrences of pulmonary complication as well as thoracic bleeding requiring reoperation, need for postoperative packed red blood cells (PRBCs) transfusion, lengths of TICU and postoperative hospital stay, and in-hospital mortality.

### 2.1 Statistical analysis

Continuous data are expressed as mean and standard deviation. Categorical data are described with percentages. Comparisons of continuous variables were performed using Student unpaired *t* test. Chi-square test was used to analyze categorical variables between the 2 groups. A *P*-value of less than .05 was considered statistically significant. All statistical analysis was performed with the use of SPPS 17.0 for Windows (SPSS Inc, Chicago, IL).

## 3 Results

The 2 groups were similar with respect to gender, age, weight, and height. There were no differences in ASA status, preoperative FVC, FEV1, type, and duration of surgery between the groups (Table 1).

**Table 1:**
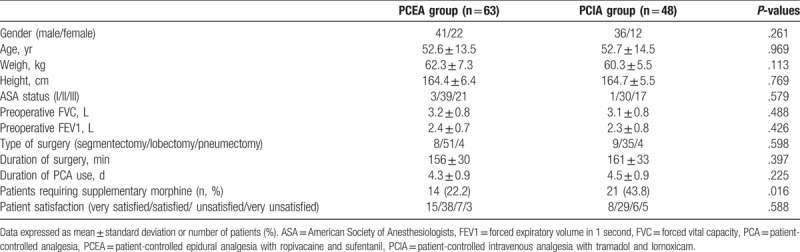
Patient characteristics, type and duration of surgery, duration of PCA use, requirement of supplementary morphine, and patient satisfaction.

**Figure 1:**
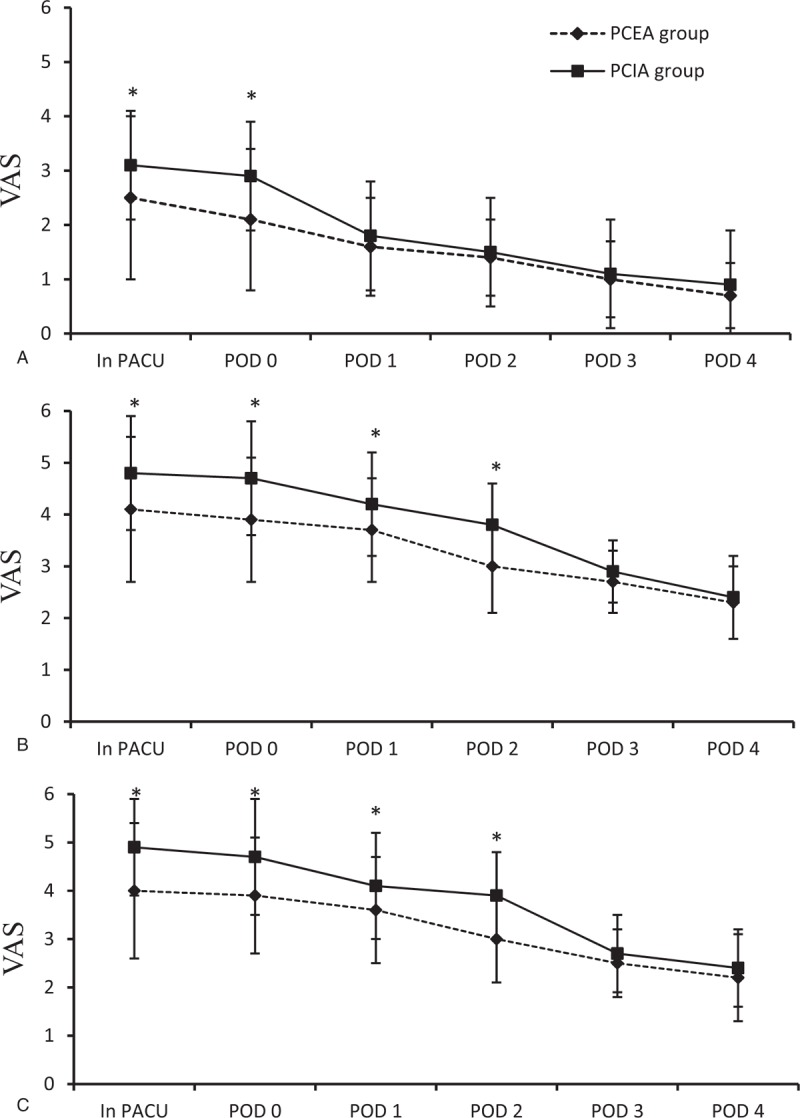
Data expressed as mean ± standard deviation. ^∗^*P* < .05, compared with the PCEA group. VAS = visual analog scale, PACU = postanesthetic care unit, POD 0 = the day of surgery; POD1, POD2, POD3, and POD 4, first, second, third, and fourth postoperative day, PCEA = patient-controlled epidural analgesia with ropivacaine and sufentanil, PCIA = patient-controlled intravenous analgesia with tramadol and lornoxicam.

As Figure 1 shows, the postoperative pain intensity decreased over time in both the PCEA group and PCIA group. Moreover, VAS scores were always below 4 at rest in the 2 groups during the 4-day postoperative period. In the PCIA group, patients reported significantly higher VAS values at rest in PACU and on the day of surgery when compared with the PCEA group. From POD1 through POD 4, pain control at rest was similar between the groups (Fig. 1A). Meanwhile, PCIA group had higher pain scores on coughing and during mobilization than the PCEA group in the first 2 days after surgery. From POD 3 to POD 4, pain scores during coughing and mobilization were comparable between the groups (Fig. 1B and C).

Supplementary morphine was required during the study period in 21 patients (43.8%) in the PCIA group and 14 patients (22.2%) in the PCEA group. There was a significant difference between the 2 groups (*P* < .05). The duration of PCA use and patients overall satisfaction with postoperative analgesia were similar in both groups (Table 1).

No significant differences in the incidence of nausea and/or vomiting, pruritus, oversedation, urinary retention, and hypotension between the groups were found. During the period of study, none of the patients had respiratory depression in either group.

As shown in Table 2, the occurrence of postoperative pulmonary complications did not differ between the PCEA and the PCIA groups. There were no differences between the groups with regard to length of TICU, length of hospital stay, and in-hospital mortality. Two deaths occurred in the PCEA group. One patient died of ventricular arrhythmias on POD 5, and the other one died on POD 7 from respiratory failure. One patient in the PCIA died from severe pneumonia on POD 11.

**Table 2:**
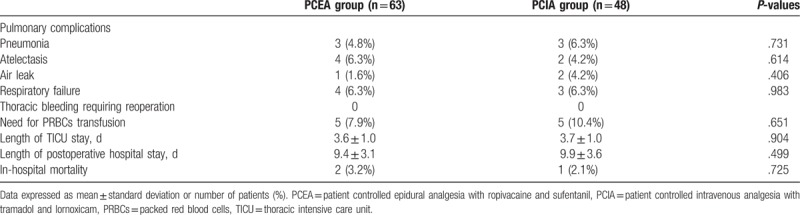
Postoperative outcomes.

## 4 Discussion

The present study suggests that although PCIA using tramadol and lornoxicam failed to provide as good pain relief as PCEA using sufentanil and ropivacaine in the early period following thoracic surgery, both analgesia modalities are effective on pain control at rest throughout the study period with similar incidences of side effects, pulmonary complication and in-hospital mortality.

Severe acute pain after thoracotomy caused by surgical incision, stretch of ligaments, and placement of rib retractors in intercostal spaces and pleural manipulation is a normal response to all these insults. Inadequate pain control produces a restrictive pattern of respiration, which may lead to hypoxemia, retention of secretions, atelectasis, pneumonia, and respiratory failure.^[14,15]^ Therefore, efficient pain relief has far-reaching consequences for short-term and long-term recovery.

There have been some studies that have evaluated the efficacy of intravenous tramadol combined with NSAIDs in post-thoracotomy pain management. Esme et al^[12]^ demonstrated that the addition of flurbiprofen to systemic tramadol resulted in reduced postoperative pain, mean additional analgesic consumption, and postoperative inflammatory response. Another study prospectively collected data to compare TEA with intravenous tramadol plus ketorolac in fifty patients after thoracic procedure.^[13]^ The authors found both analgesic techniques were able to maintain a good control of pain at rest with comparable incidence of side effects during the study period, whereas epidural analgesia showed more efficacy as for as incident pain relief.

There are several differences between our present research and previously published literature. First, both TEA and intravenous analgesia in our study were given in the form of PCA, which has been demonstrated to be able to provide better pain control with fewer adverse effects. Meanwhile, we used a low-dose background infusion in addition to PCA bolus because studies have shown this decreased the need for rescue analgesics and enhanced postoperative analgesia and patient satisfaction.^[16,17]^ Second, in PCIA group, tramadol was administered in combination with lornoxicam, which is an NSAID that blocks constitutive cyclooxygenase-1 (COX-1) and inducible cyclooxygenase-2 (COX-2) and consequently inhibits the production of prostaglandins and reduces the development of sensitization process. Compared with other oxicams, lornoxicam has high-efficiency potential, good gastrointestinal tolerance, and a short plasma half-life of 3 to 5 hours. Moreover, it has also been reported not to exert a serious nephrotoxic effect.^[18,19]^ Finally, as far as we know, this is the first study to compare the effect of PCIA using the combination of tramadol and an NSAID with PCEA on postoperative outcome.

In the present study, patients in the PCIA group reported higher pain scores on coughing and mobilization during the first 2 days after surgery when compared with the PCEA group. The possible explanation is the epidural administration of opioid provides segmental analgesia and improves the quality and duration of sensory block produced by local anesthetic.^[20,21]^

Despite improved pain control with PCEA, overall patient satisfaction did not differ between the 2 groups. This underscores that patient satisfaction is influenced by complex factors. One important reason is the similar incidence of side effects between the 2 groups. It is notable that none of our patients developed respiratory depression, which probably attributes to the concurrent administration of 2 drugs with different modes of action in both groups. The synergistic effects could reduce the total amount of each drug needed, resulting in fewer unwanted side effects.^[10,22]^

Increased risk of bleeding is one of the major concerns with perioperative NSAIDs therapy.^[23]^ In our study, there was no patient developed excessive bleeding and required reoperation in each group during the postoperative period. In addition, the percentage of patients requiring PRBCs transfusion after surgery was comparable in the 2 groups. These results were consistent with previous reports which have indicated that lornoxicam had no effect on postoperative bleeding, bleeding time, or blood transfusions requirements.^[24,25]^

Researchers have compared the outcomes between TEA and intravenous analgesia after thoracic procedure. Some strongly suggest improved outcomes with the use of TEA, whereas others have found no improvement.^[5,26–28]^ In the present study, we found the types of pain treatment did not affect the incidence of clinical respiratory complications, the length of hospital stay and in-hospital mortality. More studies are needed before definite conclusions can be drawn.

A few limitations of our study should be mentioned. First of all, we cannot exclude the possibility of selection bias for the type of analgesia due to the retrospective nature of the study. Second, postoperative FVC and FEV1 values were not collected because pulmonary function test was not reperformed after surgery in all patients, so we were not able to examine the effects of PCEA and PCIA on lung function. Last, as perioperative coagulation profiles could not be obtained in this retrospective analysis, it is impossible for the authors to evaluate accurately the risk of postoperative bleeding associated with lornoxicam use.

This retrospective study has demonstrated that PCIA with tramadol and lornoxicam can be considered as a safe and effective alternative with respect to pain control and postoperative outcomes for patients underwent thoracic surgery. Certainly, collecting data in a prospective manner is required to verify the current results.

## Author contributions

**Conceptualization:** Juying Jin, Su Min.

**Data curation:** Juying Jin, Su Min.

**Formal analysis:** Juying Jin.

**Investigation:** Juying Jin, Qibin Chen, Dong Zhang.

**Methodology:** Juying Jin, Su Min.

**Project administration:** Juying Jin, Qibin Chen, Dong Zhang.

**Resources:** Juying Jin, Su Min.

**Software:** Su Min.

**Supervision:** Su Min.

**Validation:** Qibin Chen, Dong Zhang.

**Visualization:** Qibin Chen, Dong Zhang.

**Writing – original draft:** Juying Jin.

**Writing – review and editing:** Juying Jin.
